# Solution of the Falkner–Skan wedge flow by a revised optimal homotopy asymptotic method

**DOI:** 10.1186/s40064-016-2147-z

**Published:** 2016-04-26

**Authors:** A. G. Madaki, M. Abdulhameed, M. Ali, R. Roslan

**Affiliations:** Centre for Research in Computational Mathematics Faculty of Science, Technology and Human Development, Universiti Tun Hussein Onn Malaysia, 86400 Batu Pahat, Johor Malaysia; Department of Mathematics, School of Science and Technology, Federal Polytechnic Bauchi, P.M.B. 0231, Off Dass Road, Bauchi, Nigeria

## Abstract

In this paper, a revised optimal homotopy asymptotic method (OHAM) is applied to derive an explicit analytical solution of the Falkner–Skan wedge flow problem. The comparisons between the present study with the numerical solutions using (fourth order Runge–Kutta) scheme and with analytical solution using HPM-Padé of order [4/4] and order [13/13] show that the revised form of OHAM is an extremely effective analytical technique.

## Background

In the last few decades, the problems that consist of nonlinear terms have grabbed the interest of many researchers because of its challenging to handle. Many efforts have been made by many researchers to investigate the concrete solutions to such problem of nonlinear differential equations. Due to the fact that, many nonlinear problems do not have a small parameter, so this is what has confined many analytical techniques, among which we have a perturbation technique, and other traditional methods which require the presence of a small parameter in the equation (Nayfeh and Mook 1979). We consider the two-dimensional incompressible laminar boundary layer equations, which are expressed in the form of nonlinear third-order ordinary differential equations as follows.

1 $$f^{\prime \prime \prime } + ff^{\prime \prime } + \beta \left( {1 - f^{\prime 2} } \right) = 0,$$

subject to the boundary conditions

2 $$f\left( 0 \right) = 0, \quad f^{\prime } \left( 0 \right) = 0,\quad f^{\prime } \left( { + \infty } \right) = 1.$$

This equation was first stated by Falkner and Skan (1931). Though, later on their solutions and dependency on the parameter β were investigated by Hartree (1937). The solutions of Falkner–Skan equation have been analytically investigated by many scholars. Abbasbandy and Hayat (2009) studied the solution of the magnetohydrodynamic (MHD) Falkner–Skan flow by homotopy analysis method (HAM). They found that the value of skin friction increases with the increase of magnetic field parameter, while the boundary layer thickness decreases. Yao (2009) investigated the temperature distribution in the Falkner–Skan wedge flow using HAM. Analytical solutions of momentum and heat transfer of the Falkner–Skan flow with algebraic decay have been studied by Fang et al. (2012). They observed that the value of the flow controlling parameter, *b* decreases with the decrease of the wall movement parameter, λ. Although, the value of the wall mass transfer, γ first decreases and then increases, while the wall temperature gradient increases with the decrease of pole number, *n* and the increase of Prandtl number, *Pr*. Hayat et al. (2011) studied the porous medium and mixed convection of Falkner–Skan wedge flow of a power-law fluid using HAM, their results show that dimensionless velocity distribution decreases with the increase in *Pr*. It was observed that the velocity profile increases when the Reynolds number, *Re* is increased. Yao and Chen (2009) found the series solution to the Falkner–Skan equation with stretching boundary using HAM and compared their results numerically by fourth-order Runge–Kutta method combined with Newton–Raphson technique. A good agreement was found between both approximate analytical solution and numerical result. A numerical method for the solution of the Falkner–Skan equation was investigated using a shooting method by Asaithambi (1997). Asaithambi (1997) was able to obtain the accelerating, constant, decelerating, and reverse flows numerically. The solution of the Falkner–Skan equation for wedge using Adomian decomposition method (ADM) by Alizadeh et al. (2009) indicated that the percentage of error decreases by increasing the number of the ADM terms. Rajagopal et al. (1983) investigated the Falkner–Skan boundary layer flow of a homogeneous incompressible second grade fluid past a wedge placed symmetrically with respect to the fluid flow direction. Moreover, many researchers devoted themselves on investigating the problem of Falkner–Skan flow (Kuo 2005; Pantokratoras 2006; Ishak et al. 2009; Zhu et al. 2009; Rosales-Vera and Valencia 2010; Hsiao 2011; Yacob et al. 2011; Parand et al. 2011; Abdulhameed et al. 2015).

The aim of this paper is to obtain an explicit analytical solution of the Falkner–Skan equation by using a revised OHAM which introduces another function in the auxiliary function in the original OHAM (Marinca and Herisanu 2008, 2014) which is quite good enough to handle the strong challenges in nonlinear differential equations. Recently, Herisanu et al. (2015) studied an analytical approach to non-linear dynamical model of a permanent magnet synchronous generator using OHAM. They studied four different cases at various moments of inertia and electrical resistances specific to sudden short circuit produced at the generator terminals and sudden change of load. They found very promising results when validated with numerical solutions. However, this study will be compared with the numerical solution as well as the solutions by Bararnia et al. (2012) for the acceleration flow (β > 0) and Rajagopal et al. (1983).

## Basic idea of revised OHAM (Marinca and Herisanu 2008, 2014)

Here OHAM to the differential equation as follows:

3 $$L\left( {f\left( z \right)} \right) + g\left( z \right) + N\left( {f\left( z \right)} \right) = 0,\quad B\left( {f,\frac{df}{dz}} \right) = 0$$

where $L$ is a linear operator, $z$ is an independent variable, $f\left( z \right)$ is an unknown function, $g\left( z \right)$ is a known function, $N\left( {f\left( z \right)} \right)$ is a nonlinear operator and $B$ is a boundary operator.

By means of OHAM one first constructs a family of equations:

4 $$\left( {1 - p} \right)\left[ {L\left( {\phi \left( {z,p} \right)} \right) + g\left( z \right)} \right] = H\left( {p,z} \right)\left[ {L\left( {\phi \left( {z,p} \right)} \right) + g\left( z \right) + N\left( {\phi \left( {z,p} \right)} \right)} \right],\quad B\left( {\phi \left( {z,p} \right),\frac{{\partial \phi \left( {z,p} \right)}}{dz}} \right) = 0,$$

where $p \in \left[ {0,1} \right]$ is an embedding parameter, $L$ is a linear operator which depends on the boundary operator $B$ and on the initial approximation $f_{0}$, $H\left( {p,z} \right)$ is a nonzero auxiliary function for $p \ne 0$, here $H\left( {0,z} \right) = 0$ and $\phi \left( {z,p} \right)$ is an unknown function, respectively. Obviously, when $p = 0$ or $p = 1$ it holds

5 $$\phi \left( {z,0} \right) = f_{0} \left( z \right),\quad {\text{or}}\quad \phi \left( {z,1} \right) = f\left( z \right).$$

As $p$ increases from 0 to 1, the solution $\phi \left( {z,p} \right)$ varies from $f_{0} \left( z \right)$ to the solution $f\left( z \right)$, where $f_{0} \left( z \right)$ is obtained from Eq. (4) for $p = 0$:

6 $$L\left( {f_{0} \left( z \right)} \right) + g\left( z \right) = 0,\quad B\left( {f_{0} ,\frac{{df_{0} }}{dz}} \right) = 0.$$

Instead of written the auxiliary function as $H\left( p \right) = pC_{1} + p^{2} C_{2} + {\cdots},$ we choose the auxiliary function $H\left( {p,z} \right)$ in the form

7 $$H\left( {p,z} \right) = ph_{1} \left( {z,C_{1i} } \right) + p^{2} h_{2} \left( {z,C_{2i} } \right) + \cdots + p^{m} h_{m} \left( {z,C_{mi} } \right),$$

where $h_{1} , h_{2} , \ldots ,h_{m}$ are functions depending on the variable $z$ and convergence-control parameters $C_{1i} , C_{2i} , \ldots ,C_{mi}$ for $i = 1,2, \ldots ,$ which can be determined later. Let us consider the solution of Eq. (4) is in the form

8 $$\phi \left( {z,p,h_{i} } \right) = f_{0} \left( z \right) + \mathop \sum \limits_{k \ge 1} f_{k} \left( {z,h_{i} } \right)p^{k} ,\quad i = 1,2, \ldots ,$$

Substituting Eq. (8) into Eq. (4) and equating the coefficients of like powers of $p$, we obtain the governing equation of $f_{0} \left( z \right)$ given by Eq. (6) and the governing equation of $f_{k} \left( z \right),$ as follows:

9 $$L\left( {f_{1} \left( z \right)} \right) = h_{1} N_{0} \left( {f_{0} \left( z \right)} \right),\quad B\left( {f_{1} ,\frac{{df_{1} }}{dz}} \right) = 0,$$

$$L\left( {f_{k} \left( z \right) - f_{k - 1} \left( z \right)} \right) = h_{k} N_{0} \left( {f_{0} \left( z \right)} \right) + \mathop \sum \limits_{i = 1}^{k - 1} h_{i} \left[ {L\left( {f_{k - i} \left( z \right)} \right) + N_{k - i} \left( {f_{0} \left( z \right),f_{1} \left( z \right), \ldots ,f_{k - i} \left( z \right)} \right)} \right],$$

10 $$B\left( {f_{k} ,\frac{{df_{k} }}{dz}} \right) = 0,\quad k = 2,3, \ldots ,$$

where $N_{m} \left( {f_{0} \left( z \right),f_{1} \left( z \right), \ldots ,f_{m} \left( z \right)} \right)$ is the coefficient of $p^{m}$, obtained by expanding $N\left( {\phi \left( {z,p,h_{i} } \right)} \right)$ in series with respect to the embedding parameter $p$:

11 $$N\left( {\phi \left( {z,p,h_{i} } \right)} \right) = N_{0} \left( {f_{0} \left( z \right)} \right) + \mathop \sum \limits_{k \ge 1} N_{k} \left( {f_{0} ,f_{1} , \ldots ,f_{k} } \right)p^{k} ,\quad i = 1,2, \ldots ,$$

where $\phi \left( {z,p,h_{i} } \right)$ is given by Eq. (8).

It should be emphasized that $f_{k}$ for integer $k \ge 0$ are governed by the linear Eqs. (6), (9), and (10) with the linear boundary conditions that came from origin problem, which can be solved easily.

The convergence of the series (8) depends upon the auxiliary functions $h_{1} , h_{2} , \ldots$. If it is convergent at $p = 1$, one has

12 $$f\left( {z,h_{i} } \right) = f_{0} \left( z \right) + \mathop \sum \limits_{k \ge 1} f_{k} \left( {z,h_{i} } \right).$$

Generally, the solution of Eq. (3) can be determined approximately in the form:

13 $$f^{\left( m \right)} \left( {z,h_{i} } \right) = f_{0} \left( z \right) + \mathop \sum \limits_{k = 1}^{m} f_{k} \left( {z,h_{i} } \right),\quad i = 1,2, \ldots ,m.$$

Substituting Eq. (13) into Eq. (3) it gives the following residual

14 $$R\left( {z,h_{i} } \right) = L\left( {f^{\left( m \right)} \left( {z,h_{i} } \right) + g\left( z \right) + N\left( {f^{\left( m \right)} \left( {z,h_{i} } \right)} \right)} \right),\quad i = 1,2, \ldots ,m.$$

If $R\left( {z,h_{i} } \right) = 0$, then $f^{\left( m \right)} \left( {z,h_{i} } \right)$ happens to be the exact solution. However, such case will not occur for problems that consists of nonlinear, though we can minimize the functional

15 $$J\left( {C_{ij} } \right) = \mathop \int \limits_{a}^{b} R^{2} \left( {z,h_{i} } \right)dz,\quad i = 1,2, \ldots ,m;\quad j = 1,2, \ldots$$

where $a$ and $b$ are two values, depending on the given problem. The unknown convergence-control parameters $C_{ij} ,i = 1,2, \ldots ,m$; $j = 1,2, \ldots$ can be optimally identified from the conditions

16 $$\frac{\partial J}{{\partial C_{ij} }} = 0.$$

With these convergence-control parameters known, the approximate solution (of order $m$) (13) is well-determined. Furthermore, the convergence-control parameters $C_{ij}$ can be obtained using the methods such as Galerkin, Ritz, least square or collocation.

It is easy to observe that so-called homotopy perturbation method (HPM) is a special case of Eq. (4) when $H\left( p \right) = - p$, and on the other hand, HAM is another special case of Eq. (4) when $H\left( p \right) = - ph$ (where the parameter $h$ is chosen from so-called $h$-curves), and they can all be used to determine the parameters $C_{ij}$. While an important feature of the OHAM is that using Eq. (16), a minimization of errors is obtained.

## Analytical solution of Falkner–Skan equation

The auxiliary function $H\left( {p,z} \right)$, can be chosen as many ways as possible. Using the conditions given in (2), we choose the initial approximation $f_{0} \left( z \right)$ as

17 $$f_{0} \left( z \right) = z - \frac{1}{\beta k} + \frac{1}{\beta k}e^{ - \beta kz} ,$$

where $k$ is a positive convergence parameter. Then from Eqs. (2), (6) and (17), the linear operator $L$ was chosen as follows:

18 $$L\left( {f\left( z \right)} \right) = f^{\prime \prime \prime } + kf^{\prime \prime } .$$

while, the nonlinear operator $N$ takes the form:

19 $$\begin{aligned} N\left( {z,p} \right) & = f_{0}^{\prime \prime \prime } + f_{0} f_{0}^{\prime \prime } + \beta \left( {1 - f_{0}^{\prime 2} } \right) + p\left[ {f_{1}^{\prime \prime \prime } + f_{0} f_{1}^{\prime \prime } + f_{1} f_{0}^{\prime \prime } - 2\beta \left( {f_{0}^{\prime } f_{1}^{\prime } } \right)} \right] \\ & \quad + p^{2} \left[ {f_{2}^{\prime \prime \prime } + f_{0} f_{2}^{\prime \prime } + f_{1} f_{1}^{\prime \prime } + f_{2} f_{0}^{\prime \prime } - 2\beta \left( {f_{0}^{\prime } f_{2}^{\prime } + f_{1}^{\prime 2} } \right)} \right] + \cdots \\ \end{aligned}$$

From Eq. (19) we equate the coefficients of like powers of $p$ to obtain

20 $$p^{0} : N_{0} \left( {f_{0} } \right) = f_{0}^{\prime \prime \prime } + f_{0} f_{0}^{\prime \prime } + \beta \left( {1 - f_{0}^{\prime 2} } \right),$$

21 $$p^{1} : N_{1} \left( {f_{0} ,f_{1} } \right) = f_{1}^{\prime \prime \prime } + f_{0} f_{1}^{\prime \prime } + f_{1} f_{0}^{\prime \prime } - 2\beta \left( {f_{0}^{\prime } f_{1}^{\prime } } \right),$$

22 $$p^{2} : N_{2} \left( {f_{0} ,f_{1} ,f_{2} } \right) = f_{2}^{\prime \prime \prime } + f_{0} f_{2}^{\prime \prime } + f_{1} f_{1}^{\prime \prime } + f_{2} f_{0}^{\prime \prime } - 2\beta \left( {f_{0}^{\prime } f_{2}^{\prime } + f_{1}^{\prime 2} } \right).$$

Now substituting Eq. (17) into Eq. (20), we obtain:

23 $$N_{0} \left( {f_{0} } \right) = \left( {k\beta z - k^{2} \beta^{2} + 2\beta - 1} \right)e^{ - k\beta z} + \left( {1 - \beta } \right)e^{ - 2k\beta z} .$$

Choosing $m = 1$ into Eq. (7) and by applying Eq. (23), the auxiliary function $H\left( {p,z} \right)$ can be written in the form, $H\left( {p,z} \right) = ph_{1} \left( {z,C_{1i} } \right)$ where $ph_{1} \left( {z,C_{1i} } \right)$ is chosen in such a way that $N_{0}$ and $N_{0} h_{1}$ can be in the same form, and hence we can consider:

24 $$H\left( {p,z} \right) = p\left( {C_{11} + C_{12} e^{ - k\beta z} + C_{13} e^{ - 2k\beta z} } \right),$$

where $C_{11} , C_{12} , C_{13}$ and $k$ are convergence-control parameters to be determined.

Here, Eq. (9) becomes:

$$f_{1}^{\prime \prime \prime } \,+ \,kf_{1}^{\prime \prime } = \left( {C_{11} + C_{12} e^{ - k\beta z} + C_{13} e^{ - 2k\beta z} } \right)\left[ {\left( {k\beta z - k^{2} \beta^{2} + 2\beta - 1} \right)e^{ - k\beta z} + \left( {1 - \beta } \right)e^{ - 2k\beta z} } \right],$$

25 $$f_{1} \left( 0 \right) = f_{1}^{{\prime }} \left( 0 \right) = f_{1}^{{\prime }} \left( \infty \right) = 0.$$

From now on, for simplification we will write $C_{1} ,C_{2}$ and $C_{3}$ instead of $C_{11} , C_{12}$ and $C_{13}$, respectively.

Now the solution of Eq. (25) takes the form:

26 $$f_{1} \left( z \right) = \frac{1}{{108k^{3} \left( {k^{3} - 12} \right)}}\left[ \begin{aligned} - 1296k^{5} C_{1} - 324k^{5} C_{2} - 144k^{5} C_{3} + 3888k^{3} C_{1} + 648k^{3} C_{2} + 240k^{3} C_{3} \hfill \\ + \left( { - 6k^{4} zC_{3} + 72kzC_{3} + 6k^{5} C_{3} - 13k^{3} C_{3} - 72k^{2} C_{3} + 156C_{3} } \right)e^{ - 3kz} \hfill \\ \end{aligned} \right]$$

The first-order approximate solution (13) is

27 $$f^{\left( 1 \right)} \left( z \right) = f_{0} \left( z \right) + f_{1} \left( z \right) .$$

Substituting Eqs. (17) and (26) into Eq. (27) we obtain:

28 $$f^{\left( 1 \right)} \left( z \right) = \frac{1}{{108k^{3} \left( {k^{3} - 12} \right)}}\left[ \begin{aligned} - 1296k^{5} C_{1} - 324k^{5} C_{2} - 144k^{5} C_{3} + 3888k^{3} C_{1} + 648k^{3} C_{2} + 240k^{3} C_{3} \hfill \\ + \left( { - 6k^{4} zC_{3} + 72kzC_{3} + 6k^{5} C_{3} - 13k^{3} C_{3} - 72k^{2} C_{3} + 156C_{3} } \right)e^{ - 3kz} \hfill \\ \end{aligned} \right] + z - \frac{1}{\beta k} + \frac{1}{\beta k}e^{ - \beta kz}$$

The residual (14) becomes in this case:

29 $$R\left( {z,C_{1} ,C_{2} ,C_{3} ,k} \right) = f^{{(1)}^{\prime \prime \prime}} \left( z \right) + f^{(1)} \left( z \right)f^{{(1)}^{\prime \prime}} \left( z \right) + \beta \left( {1 - f^{{{{(1)}^{\prime} }^2}} \left( z \right)} \right).$$

From Eq. (16), this becomes

$$\frac{\partial J}{{\partial C_{i} }} = \frac{\partial J}{\partial k} = 0,\quad i = 1,2,3$$ we obtain:

30 $$C_{1} = 0.00186322166, \quad C_{2} = 0.4026020376,\quad C_{3} = - 0.02385423487,\quad k = 1.231562651244.$$

**Fig. 1:**
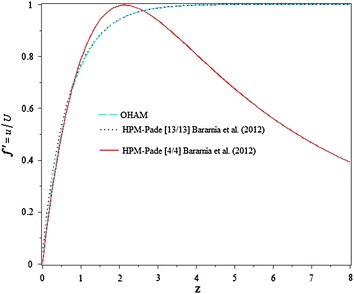
Comparison of OHAM solution with HPM-Padé solution and the effect of increasing the order of its approximation, on the velocity profile for $\beta = 1.$

**Fig. 2:**
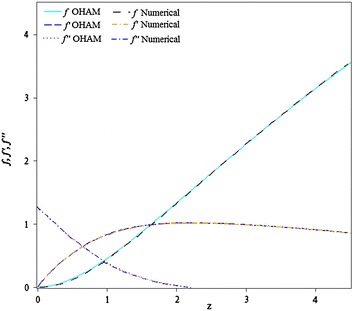
Comparison of OHAM with numerical solutions for $\beta = 1.$

Sequel to the presentations made in Figs. 1 and 2, where the results for this study appears perfectly matched that of Bararnia et al. (2012) from which the increasing order of Padé approximation on the velocity profile shows a significant effect, as depicted in Fig. 1. While Fig. 2, has indicated the good agreements between the present studies with numerical solution.

## Conclusions

In this study, a new analytical technique is proposed to get a solution of Falkner–Skan equation. The method gives a desired analytical solution of the 2D laminar, incompressible viscous fluid flow over a semi-infinite wedge. The validation of this study with numerical and Bararnia et al. (2012), as shown in Figs. 1 and 2, show the excellence and capability of OHAM towards handling both linear and nonlinear problems. However, an excellent agreement between those methods has achieved, because the values we obtained for $f^{\prime \prime } \left( 0 \right) = 1.23140046$ at $\beta = 1$, agreed with $f^{\prime \prime } \left( 0 \right) = 1.23150$ (Bararnia et al. 2012) and $f^{\prime \prime } \left( 0 \right) = 1.2325$ (Rajagopal et al. 1983) all at $\beta = 1$. Despite the fact that only first-order approximation been generated.
